# Causes of abortion in Iranian sheep flocks and associated risk factors

**DOI:** 10.1007/s11259-022-09986-5

**Published:** 2022-09-06

**Authors:** Hossein Esmaeili, Amir Pasha Shakeri, Zia Nosrati Rad, Ehsan Baghal Arani, Sergio Villanueva-Saz, Héctor Ruiz, Delia Lacasta

**Affiliations:** 1 Department of Microbiology and Immunology, Faculty of Veterinary Medicine, University of Tehran, Tehran, Iran; 2 Department of Animal Pathology, Veterinary Faculty, University of Zaragoza, 50013 Zaragossa, Spain; 3 Instituto Agroalimentario de Aragón-IA2, Universidad de Zaragoza-CITA, Zaragoza, Spain

**Keywords:** Abortion, Iran, Risk factors, Sheep, Small ruminants

## Abstract

Abortion is a major issue in sheep husbandry. It can result in significant economic losses and a severe public health risk. This survey assessed the infectious and non-infectious causes of abortion in Iranian sheep flocks and determined the main risk factors. In this cross-sectional survey, causes of abortion were evaluated in 757 sheep flocks, and risk factors were analysed. A checklist containing general animal information for each abortion outbreak evaluated was filled in. Data were analysed using univariate tests and multivariable binary logistic regression analysis. In this sense, parity, gestational age of the aborted fetus, vaccination protocol, mineral supplementation and history of stillbirth showed significant associations with abortion. Infectious agents such as *Coxiella burnetti* (22.7%), *Chlamydia abortus* (12.3%) and *Brucella melitensis* (10.4%) were the most frequently isolated in the investigated flocks, with more than 2% of abortion rates. On the other hand, non-infectious agents such as trauma, pregnancy toxaemia and vitamin E/Se deficiency were involved in those flocks with low abortion rates (less than 10%). Results revealed multiple causes of abortion outbreaks among Iranian sheep flocks, which need careful investigation to identify possible aetiology and risk factors. Further studies are necessary to evaluate if these factors are similar to other countries in the same region.

## Introduction

Small ruminants have a significant role in the Iranian agricultural economy and perform as leading actors in the field of the livelihoods of nomadic and rural farmers (Esmaeili and Hamedi 2018). Iran has more than 30 indigenous breeds of sheep with a population of 45 million heads (Ministry of agriculture 2020). The primary purpose of the Iranian breeds is meat production, which leads to an annual production of 265,000 tons (Ministry of agriculture 2020). Mutton meat is essential in food self-sufficiency and supplies a sound animal protein source. Milk is also a vital product for Iranian sheep, producing 252,000 tons/year, providing for a large part of the population (Ministry of agriculture 2020). Due to these reasons, sheep abortions severely affect food production, animal health and the agricultural economy. In addition, its zoonotic potential can pose a serious risk to public health.

According to Iran Veterinary Organization (IVO) protocols, Iranian sheep health plans include vaccination against brucellosis, sheep pox, Peste des Petits Ruminants (PPR), anthrax, Foot-and-mouth disease (FMD), contagious agalactia and enterotoxemia. However, the last two are not mandatory (Esmaeili et al. 2017).

Abortions can result in significant economic losses and severe risks to public health. Financial losses are associated with both direct and indirect costs. Some of these indirect costs are related to an increase in lambing intervals, which supposes more food expenses, a detriment in the incomes of sold muttons, fewer replacement lambs available and an increase in veterinary treatments (Pugh and Baird 2012).

In most sheep flocks, the percentage of ewes visibly aborting is less than 2% (Menzies 2011), being this considered a normal rate. Therefore, in the present study, an abortion outbreak was considered when the total percentage of abortions exceeded 2%.

There are various potential agents underlying the causes of abortion, which can mainly be classified into infectious and non-infectious. Non-infectious causes remain unknown in most abortions because they do not produce a high percentage of aborted ewes and therefore are not studied in depth. Infectious causes, which are more rapidly diagnosed, are more critical (Njaa 2011). However, several non-infectious aetiologies can be associated with abortions in sheep, such as traumatism, pregnancy toxaemia, vitamin E/Se deficiency, stressful handling, overcrowding, or even consuming toxic plants (Youngquist and Threlfall 2006; Pugh and Baird 2012). Non-infectious causes are usually related to a low rate of abortion. However, infectious aetiology is mainly associated with severe abortion outbreaks with relevant human health implications (Noakes et al. 2018). In sheep, the major infectious causes of abortion are bacteriological agents, such as *Campylobacter* spp, *Brucella melitensis*, *Listeria* spp, *Salmonella* spp*, Coxiella burnetii*, and *Chlamydia abortus* (Youngquist and Threlfall 2006; Pugh and Baird 2012). Furthermore, other infectious causes of viral origin include Border disease virus, Bluetongue and Schmallenberg virus. Likewise, parasites such as *Toxoplasma gondii* or *Neospora caninum* are common pathogens implicated in fetal losses (Youngquist and Threlfall 2006; Pugh and Baird 2012).

Significant differences exist in the prevalence of different infectious agents in other countries. Thus, in research conducted in England by the Veterinary Investigation Diagnosis Analysis (VIDA) between 2011 and 2018, bacteriological agents were the leading cause of abortion, being *C. abortus* (45%) the most frequently isolated agent (Carson 2018). In general, repeated surveys carried out in Europe have indicated *C. abortus* as a significant abortifacient agent in small ruminants (Navarro et al. 2009), although other pathogens can emerge occasionally. In southern European countries, as well as in many Asian and African regions, *B. melitensis* is also a significant pathogen in small ruminant farms (Lacasta et al. 2015). In contrast, in New Zealand, *Campylobacter* spp. and *Salmonella brandenburg* are the primary microbial abortifacient agents, whilst *C. abortus* and *C. burnetii* infections have never been diagnosed in that country (West 2002). Likewise, *Campylobacter* spp. is a significant cause of small ruminant abortion also in North America (Hazlett et al. 2013).

In Iran, an abortion research survey conducted by Hamedi et al. in 2020 found that 23.5% of abortions in sheep and goats were caused by *C. abortus* (Hamedi et al. 2020). Another survey performed in southern Iran detected *C. abortus, C. burnetii* and *Mycoplasma agalactiae* as the main abortion agents in aborted fetuses (Heidari et al. 2018). In Ethiopia, Gebretensay et al. (2019), reported that up to 20% of sheep flocks had recent abortion outbreaks, obtaining 89.0% of seropositive results for *Chlamydia* spp., 68.3% for *C. burnetti* and 70.8% for *T. gondii* (Gebretensay et al. 2019).

The reproductive performance should be improved in sheep flocks in order to increase the production of lamb and other products. Thus, identifying the causes of abortion and their risk factors is crucial. The present survey aimed to identify the most common causes of sheep abortion and its risk factors in Iranian sheep flocks.

## Materials and methods

### Design and case selection

From 2015 to 2019, a cross-sectional survey was performed on 757 sheep flocks in Iran. The sheep breeds included in the research were local breeds such as Ghezel, Afshar, Zandi, Shall, Lori, Arabi, Torki-Ghashghaei, Bakhtiyari, Sanjabi, Moghani, Zel, Sangsari and Naeini.

During the lambing season, flocks were under active surveillance for abortion, and all flocks in this survey were visited and examined by a veterinarian. The percentage of abortions was calculated based on the number of pregnant ewes, and each flock was sampled once. After compiling the clinical history provided by the farmer, the aborted ewes were examined by the veterinarian and the collected samples were sent to the laboratory with ice (-20ºC). There, the fetuses were necropsied and sampled.

General information about each flock and affected animals was recorded (Table 2). Each item was dichotomised to either zero (for no) or one (for yes). Since the abortion rate of less than 2% is normal in sheep flocks, these flocks were considered control.

A case was defined as one or more fetuses or tissues submitted to a microbiology laboratory simultaneously from a single flock. Cases consisting of intact fetuses or tissues from necropsies conducted in the field only were included if all the following fresh tissues were available for evaluation: brain, heart, lung, liver, kidneys and abomasal contents. Abortion work-ups included evaluation of clinical history and necropsy findings. Gestational age was estimated using crown-rump length measurements at the time of necropsy (Noden and Lahunta, 1985).

### Sampling

Before collecting blood, the sheep's wool was trimmed around the jugular region. Blood sampling consisted of collecting aseptically 2 ml of blood by jugular venepuncture, with the collected volume divided equally between a sterile blood collection tube (to obtain the serum for BHB analysis) and a second tube containing ethylenediaminetetraacetic acid (EDTA) anticoagulant (for PCR analysis of BTV). Blood and separated serum were stored at − 20 °C until processing.

Blood samples were taken only from ewes with clinical signs of pregnancy toxaemia (hind limbs oedema or thin ewes that aborted twins) and those with blue tongue symptoms (bloody discharge from the nose and mouth, swelling and oedema of the lips and gums, swollen and purple tongue). Stomach content, brain, heart and lung of aborted fetuses were taken aseptically for culture and molecular detection of infectious agents.

### Bacteriological culture

The samples of aborted fetuses were inoculated on Blood agar and MacConkey agar (Merck, Germany) in order to isolate bacteria that were cultivable on routine media cultures. A pair of plates were incubated aerobically, and the other pair anaerobically for 48 h at 37 °C. The plates were inspected for bacterial growth after 16–20 h of cultivation and after an additional 24 h in cases of no or slow bacterial growth at first inspection. The selection of colonies for subculturing was based on colony morphology and the number of colony types that were not considered post-mortem contamination. Colonies were subcultured and identified using standard methods for phenotypical characterisation (Koneman et al. 1997).

"Other bacteria" were defined as agents other than *Campylobacter* spp*., Salmonella* spp*., C. abortus, Mycoplasma* spp*. B.melitensis, Trueperella pyogenes*, *Listeria monocytogenes, E. coli* or *C. burnetii*. Mixed cultures containing fewer than three distinct bacterial colony types with no confirmed pathogenic bacteria were not considered positive for an etiologic agent.

### Mycologic examination

A sterile disposable syringe was used to aseptically collect l-3 ml of abomasal content to diagnose mycotic infections. In addition, portions of the lung, eyelid, and skin (if there was evidence of dermatitis) were collected depending on the availability of fetal tissues. A sterile scalpel was used to scrape a small amount of material from any fetal skin with gross lesions. This material, fetal lung that had been macerated with a sterile mortar and pestle, and 0.2 ml of fetal abomasal content were separately spread onto the surface of plates of Sabouraud dextrose agar (SDA) containing 1,000 units/ml of penicillin G. Solid media were incubated at both room temperature, and 37° C. Plates were examined daily for the first week and twice each week for the next two weeks. Portions of detectable mycotic growth were transferred to potato dextrose agar (PDA) and SDA plates and slants. PDA was used as a growth medium for slide cultures, incubated for 3–7 days and then stained with lactophenol cotton blue. Filamentous isolates were identified from gross and microscopic characteristics (Beneke and Rogers. 1971). Fungal diagnoses included visualising disseminated hyphal elements or yeast with or without positive cultures from nonplacental sites (to remove bias from possible environmental contamination).

### Molecular analysis

According to the manufacturers' instructions, DNA/ RNA was extracted from 25 mg of tissue samples using a DNA/RNA extraction kit (High Pure PCR Template Preparation Kit, Roche Company, Germany), and diagnosis of each abortion agent was performed using the PCR assays as described previously in the literature: *Mycoplasma* spp. (Van Kuppeveld et al., 1992), *Trueperella pyogenes* (Ülbegi-Mohyla et al., 2010), Border disease (Vilček and Paton, 2000), Bluetongue (Anthony et al., 2007), *Campylobacter* spp. (Bang et al., 2001), *Toxoplasma gondii* (Burg et al., 1989), *Brucella melitensis* (Bricker and Halling, 1994), *Listeria monocytogenes* (Gouws and Liedemann, 2005), *Coxiella burnetii* (Hoover et al., 1992), *Salmonella* spp. (Rahn et al., 1992), *Chlamydia abortus* (Laroucau et al., 2001).

### Pregnancy toxaemia

Beta-hydroxy butyrate was measured in serum samples using the Williamson-Mellanby enzymatic method (Commercial kit, Biorex Fars Company, Shiraz, Iran) and animals that showed a BHB concentration greater than three mmol/l were considered suffering from pregnancy toxaemia (Radostits and Gay 2017).

### Final diagnosis

The final diagnosis was based on characteristic gross changes detected at necropsy and the diagnostic test results. Criteria for trauma included full-term lambs with head haemorrhage, rib fractures, intrathoracic haemorrhage, and/or ruptured liver, with no detection of infectious agents in any examined tissues. Vitamin E/selenium deficiency was diagnosed following the method described by Liu et al. (Liu et al., 1996). Finally, congenital defects were determined by fetus examination.

### Statistical analysis

The data were analysed using IBM.SPSS 25 (SPSS Inc., Chicago, IL, USA). Categorical data were presented as frequency (percentage). Univariate analysis of categorical data was conducted using the chi-square test. In univariate analyses, a multivariate binary logistic regression model included the significant variables at p values below 0.2. The results were presented as odds ratio (OR) and 95% confidence interval (CI). P values below 0.05 were considered statistically significant. Sheep flocks with a < 2% abortion rate were considered control and compared with those flocks with more than 2% abortion rate.

## Results

Over the 757 studied flocks, two hundred and thirteen (213/757: 28.1%) had an abortion rate below 2%, which, following the literature (Menzies 2011), was considered normal in a sheep flock. The remaining 544 flocks (544/757: 71.8%) had pathological abortion rates (> 2%). The final cause of the abortion was determined in 301 of the 544 abortion outbreaks analysed (55.3%).

As shown in Table 1, infectious agents were diagnosed as a cause of abortion in 287 flocks (287/757: 37.9%). Infectious agents were isolated in 2.8% (6/213) of the flocks with an abortion rate lower than 2% and 51.6% (281/544) of the flocks with an abortion rate higher than 2%. The leading infectious agent diagnosed in the survey was *Chlamydia abortus* which was diagnosed in 67 outbreaks (67/544: 12.3%), followed by *Brucella melitensis* (57/544: 10.4%), *Coxiella burnetti* (26/544: 4.8%), *Salmonella* spp. (24/544: 4.4%), *Campylobacter* spp. (20/544: 3.7%) *Mycoplasma* spp. (13/544: 2.4%), *Escherichia coli* (11/544: 2%), *Bluetongue* virus (10/544: 1.9%), *Fungi.* (11/757: 1.5%), *Aspergillus fumigatus* (8/544: 1.5%)*, Candida albicans* (3/544: 0.6%) and *Listeria* spp*.* (7/544: 1.3%).

**Table 1:** Infectious and non-infectious causes of abortion in sheep flocks in Iran are distributed according to the percentage of abortion rate

	** < 2%, N = 213 (28.1%)**^**1**^	**2–4.9%, N = 84 (11.1%)**^**1**^	**5–9.9%, N = 195 (25.8%)**^**1**^	**10–19.9%, N = 130 (17.2%)**^**1**^	**20–29.9%, N = 50 (6.6%)**^**1**^	**30–50%, N = 55 (7.3%)**^**1**^	** > 50%, N = 30 (4.0%)**^**1**^	** > 2%, N = 544 (71.9%)**^**1**^	**Overall N = 757**	**p-value**^**2**^
**Median** ± **SD / Range**	**0.87 ± 0.47 / 1.7**	**3.6 ± 0.81 / 2.9**	**7.2 ± 1.41 / 4.9**	**13.45 ± 3.43 / 9.9**	**24.64 ± 3.59 / 9.9**	**42 ± 6.97 / 20**	**58.2 ± 9.2 / 34**	**9.7 ± 15.88 / 83**		
**Infectious agents**										< 0.001
Negative	207 (97%)	60 (71%)	121 (62%)	53 (41%)	18 (36%)	8 (15%)	3 (10%)	263 (48.4%)	470 (62%)	
Positive	6 (2.8%)	24 (29%)	74 (38%)	77 (59%)	32 (64%)	47 (85%)	27 (90%)	281 (51.6%)	287 (38%)	
**Diagnosis reached**										< 0.001
No	198 (93%)	43 (51%)	118 (61%)	53 (41%)	18 (36%)	8 (15%)	3 (10%)	243 (44.7%)	441 (58%)	
Yes	15 (7.0%)	41 (49%)	77 (39%)	77 (59%)	32 (64%)	47 (85%)	27 (90%)	301 (55.3%)	316 (42%)	
***Coxiella burnetti***										> 0.05
Negative	213 (100%)	84 (100%)	185 (95%)	118 (91%)	46 (92%)	55 (100%)	30 (100%)		731 (97%)	
Positive	0 (0%)	0 (0%)	10 (5.1%)	12 (9.2%)	4 (8.0%)	0 (0%)	0 (0%)	26 (4.8%)	26 (3.4%)	
***Chlamydia abortus***										> 0.05
Negative	213 (100%)	84 (100%)	192 (98%)	120 (92%)	41 (82%)	30 (55%)	10 (33%)		690 (91%)	
Positive	0 (0%)	0 (0%)	3 (1.5%)	10 (7.7%)	9 (18%)	25 (45%)	20 (67%)	67 (12.3%)	67 (8.9%)	
***Brucella melitensis***										> 0.05
Negative	213 (100%)	84 (100%)	188 (96%)	119 (92%)	40 (80%)	33 (60%)	23 (77%)		700 (92%)	
Positive	0 (0%)	0 (0%)	7 (3.6%)	11 (8.5%)	10 (20%)	22 (40%)	7 (23%)	57 (10.4%)	57 (7.5%)	
***Chlamydia & Brucella***										0.005
Negative	213 (100%)	84 (100%)	195 (100%)	125 (96%)	49 (98%)	55 (100%)	30 (100%)		751 (99%)	
Positive	0 (0%)	0 (0%)	0 (0%)	5 (3.8%)	1 (2.0%)	0 (0%)	0 (0%)	6 (1.1%)	6 (0.8%)	
***C.burnetti & C.abortus***										< 0.001
Negative	213 (100%)	84 (100%)	195 (100%)	127 (98%)	46 (92%)	55 (100%)	30 (100%)		750 (99%)	
Positive	0 (0%)	0 (0%)	0 (0%)	3 (2.3%)	4 (8.0%)	0 (0%)	0 (0%)	7 (1.3%)	7 (0.9%)	
***Campylobacte*r spp**.										< 0.001
Negative	213 (100%)	84 (100%)	188 (96%)	119 (92%)	48 (96%)	55 (100%)	30 (100%)		737 (97%)	
Positive	0 (0%)	0 (0%)	7 (3.6%)	11 (8.5%)	2 (4.0%)	0 (0%)	0 (0%)	20 (3.7%)	20 (2.6%)	
***Salmonella* spp.**										> 0.05
Negative	213 (100%)	84 (100%)	186 (95%)	117 (90%)	48 (96%)	55 (100%)	30 (100%)		733 (97%)	
Positive	0 (0%)	0 (0%)	9 (4.6%)	13 (10%)	2 (4.0%)	0 (0%)	0 (0%)	24 (4.4%)	24 (3.2%)	
***Mycoplasma* spp**.										0.006
Negative	213 (100%)	84 (100%)	186 (95%)	126 (97%)	50 (100%)	55 (100%)	30 (100%)		744 (98%)	
Positive	0 (0%)	0 (0%)	9 (4.6%)	4 (3.1%)	0 (0%)	0 (0%)	0 (0%)	13 (2.4%)	13 (1.7%)	
***Escherichia coli***										0.082
Negative	210 (99%)	80 (95%)	188 (96%)	130 (100%)	50 (100%)	55 (100%)	30 (100%)		743 (98%)	
Positive	3 (1.4%)	4 (4.8%)	7 (3.6%)	0 (0%)	0 (0%)	0 (0%)	0 (0%)	11 (2%)	14 (1.8%)	
***Listeria* spp.**										0.046
Negative	213 (100%)	81 (96%)	191 (98%)	130 (100%)	50 (100%)	55 (100%)	30 (100%)		750 (99%)	
Positive	0 (0%)	3 (3.6%)	4 (2.1%)	0 (0%)	0 (0%)	0 (0%)	0 (0%)	7 (1.3%)	7 (0.9%)	
***Trueperella pyogenes***										< 0.001
Negative	213 (100%)	79 (94%)	195 (100%)	130 (100%)	50 (100%)	55 (100%)	30 (100%)		752 (99%)	
Positive	0 (0%)	5 (6.0%)	0 (0%)	0 (0%)	0 (0%)	0 (0%)	0 (0%)	5 (0.9%)	5 (0.7%)	
**Other bacteria**										0.029
Negative	213 (100%)	80 (95%)	189 (97%)	128 (98%)	50 (100%)	55 (100%)	30 (100%)		745 (98%)	
Positive	0 (0%)	4 (4.8%)	6 (3.1%)	2 (1.5%)	0 (0%)	0 (0%)	0 (0%)	12 (2.2%)	12 (1.6%)	
***Toxoplasma gondii***										0.2
Negative	213 (100%)	82 (98%)	193 (99%)	130 (100%)	50 (100%)	55 (100%)	30 (100%)		753 (99%)	
Positive	0 (0%)	2 (2.4%)	2 (1.0%)	0 (0%)	0 (0%)	0 (0%)	0 (0%)	4 (0.7%)	4 (0.5%)	
**Bluetongue**										0.019
Negative	213 (100%)	84 (100%)	191 (98%)	124 (95%)	50 (100%)	55 (100%)	30 (100%)		747 (99%)	
Positive	0 (0%)	0 (0%)	4 (2.1%)	6 (4.6%)	0 (0%)	0 (0%)	0 (0%)	10 (1.9%)	10 (1.3%)	
**Border Disease**										0.2
Negative	213 (100%)	84 (100%)	191 (98%)	130 (100%)	50 (100%)	55 (100%)	30 (100%)		753 (99%)	
Positive	0 (0%)	0 (0%)	4 (2.1%)	0 (0%)	0 (0%)	0 (0%)	0 (0%)	4 (0.8%)	4 (0.5%)	
**Fungi spp**.										0.014
Negative	210 (99%)	78 (93%)	193 (99%)	130 (100%)	50 (100%)	55 (100%)	30 (100%)		746 (99%)	
Positive	3 (1.4%)	6 (7.1%)	2 (1.0%)	0 (0%)	0 (0%)	0 (0%)	0 (0%)	8 (1.5%)	11 (1.5%)	
**Congenital defects**										0.2
Negative	212 (100%)	82 (98%)	195 (100%)	130 (100%)	50 (100%)	55 (100%)	30 (100%)		754 (100%)	
Positive	1 (0.5%)	2 (2.4%)	0 (0%)	0 (0%)	0 (0%)	0 (0%)	0 (0%)	2 (0.4%)	3 (0.4%)	
**Pregnancy toxaemia**										< 0.001
Negative	212 (100%)	76 (90%)	195 (100%)	130 (100%)	50 (100%)	55 (100%)	30 (100%)		748 (99%)	
Positive	1 (0.5%)	8 (9.5%)	0 (0%)	0 (0%)	0 (0%)	0 (0%)	0 (0%)	8 (1.5%)	9 (1.2%)	
**Trauma**										0.046
Negative	211 (99%)	79 (94%)	192 (98%)	130 (100%)	50 (100%)	55 (100%)	30 (100%)		747 (99%)	
Positive	2 (0.9%)	5 (6.0%)	3 (1.5%)	0 (0%)	0 (0%)	0 (0%)	0 (0%)	8 (1.5%)	10 (1.3%)	
**Vit E/selenium deficiency**										0.11
Negative	208 (98%)	82 (98%)	195 (100%)	130 (100%)	50 (100%)	55 (100%)	30 (100%)		750 (99%)	
Positive	5 (2.3%)	2 (2.4%)	0 (0%)	0 (0%)	0 (0%)	0 (0%)	0 (0%)	2 (0.4%)	7 (0.9%)	

^1^Statistics presented: n (%)

^2^Statistical tests performed: chi-square test of independence; Fisher's exact test

Attending to the classification of the causative agent, bacteriological agents caused abortion in 255 flocks (255/544: 46.9%), while viral agents affected 14 flocks (14/544: 2.6%), and fungal agents were diagnosed in 8 flocks (8/544: 1.5%).

Non-infectious causes such as trauma (10/757:1.3%), pregnancy toxaemia (9/757:1.2%), and Vitamin E/selenium deficiency (7/757:0.9%) were mainly identified in flocks with a low abortion rate, being diagnosed in 26 studied flocks (26/757: 3.4%).

In univariate analyses, birth, flock type, gestational age of the aborted fetus, history of Rev-1 vaccination, routine visit of veterinarian, vaccination protocol according to IVO, mineral supplementation and history of stillbirth had significant associations with abortion at p < 0.05 (Table 2). According to Multivariable binary logistic regression analysis, sheep that have not been vaccinated according to IVO protocols were at higher risk of suffering abortion than the others (OR: 0.04; 95% CI: 0.00–0.34; P = 0.009) (Table 2).

**Table 2:** Analysis of the risk factors associated with abortion in sheep flocks of Iran

		Resulted from univariate chi-square analysis		Resulted from multivariable logistic regression analysis	
**Variables**	**Categories**	**OR (95% CI)**	**p-value**	**OR (95% CI)**	**p-value**
**Birth**	Multiple	1 (Ref.)	< 0.001	1 (Ref.)	< 0.001
	One	5.86 (4.12—8.44)		11.0 (5.64- 22.6)	
**Flock Type**	Rural	1 (Ref.)	0.023	1 (Ref.)	0.6
	Nomadic	1.46 (1.06- 2.04)		1.17 (0.63- 2.18)	
**Flock size**	< 100	1 (Ref.)	> 0.9	-	-
	> 100	0.00 (0.00—0.03)		-	
**Abortion history**	No	1 (Ref.)	0.6	-	-
	Yes	0.9 (0.59- 1.40)		-	
**Gestational age of aborted fetus**	< 3 month	1 (Ref.)	< 0.001	1 (Ref.)	< 0.001
	> 3 month	2.5 (1.75- 3.58)		3.65 (1.86- 7.29)	
**History of Rev-1 vaccination**	No	1 (Ref.)	< 0.001	1 (Ref.)	0.4
	Yes	0.09 (0.04- 0.17)		0.5 (0.09- 1.99)	
**Presence of Cattle**	No	1 (Ref.)	0.8	-	-
	Yes	1.05 (0.76- 1.45)		-	
**Presence of Dog**	No	1 (Ref.)	> 0.9	-	-
	Yes	0.00 (0.00- 6.96)		-	
**Routine visit of veterinarian**	No	1 (Ref.)	< 0.001	1 (Ref.)	0.6
	Yes	0.2 (0.14- 0.28)		0.86 (0.47- 1.61)	
**Vaccination**	No	1 (Ref.)	< 0.001	1 (Ref.)	0.009
	Yes	0.01(0.00- 0.05)		0.04 (0.00- 0.34)	
**Mineral supplementation**	No	1 (Ref.)	< 0.001	1 (Ref.)	< 0.001
	Yes	0.02 (0.01- 0.03)		0.02 (0.01- 0.03)	
**History of Stillbirth**	No	1 (Ref.)	0.2	1 (Ref.)	< 0.001
	Yes	0.46 (0.14- 1.63)		0.0 (0.00- 0.03)	

## Discussion

This investigation is the first extensive epidemiological survey of the leading causes of abortion in Iranian sheep flocks. It provides insights into abortion agents and the risk factors involved. The causes of abortion depending on the percentage of abortion rate and the role of infectious and non-infectious causes in these reproductive failures are illustrated.

It was possible to determine the final cause of abortion in 55.3% of the studied cases with more than 2% of animals aborted. However, the success in diagnosing augmented as the abortion rate increased due to the more significant enrolment of the infectious agents in abortion outbreaks. Thus, in those flocks with an abortion rate higher than 30%, the final diagnosis was reached in more than 85% of the cases. In agreement with our results, in a study carried out in Canada, abortion agents were diagnosed in 90 out of 163 sheep fetuses (55%) and 66 of 96 goat fetuses (69%) (Hazlett et al. 2013). Likewise, in the Netherlands, infectious agents were detected in 48% of the ovine submissions (Van den Brom et al. 2012). A failure to diagnose an abortive process may be due to other different causes such as genetic, metabolic, hormonal, developmental disorders, or other reasons, which are often difficult or impractical to confirm in a routine diagnostic procedure. However, many fetuses for which no aetiology is detected have an infectious cause (Anderson 2007).

The risk of transmission of infectious agents in Iran is high due to the traditional rearing of lambs. There is a traditional trade of lambs younger than three weeks of age during the lambing season in Iran. These lambs, coming from different flocks, are reared together, fattened, and prepared for slaughter. Likewise, the ewes are slaughtered after lambing for meat production, and the lambs are sold to other flocks and reared by goats or ewes whose lambs had died or were aborted. Due to the higher adult weight of sheep and better carcass production than goats, some farmers sell their kids and rear orphaned lambs by does. Some other farmers buy these orphaned lambs to stimulate milk production of aborted ewes or to be raised by ewes whose lambs have died. Therefore, many lambs and kids from flocks with different health statuses are sold and distributed among other flocks, and the possibility of spreading various infectious agents increases (Esmaeili and Hamedi 2018).

In the present survey, various infectious agents of sheep abortion were identified, and in 46.9% of the cases, bacteria were the leading causes. According to reports from Veterinary Investigation Diagnosis Analysis of Britain from 2011 to 2018, the bacteria had the highest percentage among other infectious abortive agents (Carson 2018). In the present investigation, *C.abortus, B.melitensis* and *C.burnetti* were responsible for a high number of abortion outbreaks, especially with abortion rates higher than 10%. *C. abortus* has been reported as the predominant abortifacient bacterium throughout the world. Various reports from different countries showed the involvement of this bacterium in abortion storms and flocks with endemic abortion problems (OIE 2018).

Furthermore, a high prevalence of *C. abortus* (24.1%) among small ruminant flocks was previously reported in Iran (Esmaeili et al. 2015). Likewise, in previous studies developed in Iran, *B. melitensis, Campylobacter* spp*., C.abortus, T.gondii,* and *Salmonella* spp*.* were the leading causes of abortion recorded in small ruminant flocks (Behzadi Shahrbabak 2019). Similarly, another survey performed in southern Iran showed that *Brucella* spp., *Salmonella* spp.*, Campylobacter* spp. and *E.coli* were isolated in 20.5%, 19.6%, 7.5%, and 26.1% aborted fetuses in conventional culture, respectively (Firouzi 2006). *Campylobacter* spp. was isolated in 3.7% of the abortion outbreaks in our survey. In this sense, 9.1% and 1.5% of sheep fetuses in the Tabriz region in Iran were positive for *C. fetus* subsp*. fetus* and *C. jejuni* using PCR (Fallah et al. 2014).

Q fever has been reported among livestock and human populations in Iran and has a high seroprevalence. It has also been detected in milk, and aborted fetuses and seroprevalence among sheep flocks was reported 24.7% (Mobarez et al.^2017^). *Mycoplasma* spp. was detected in 1.7% of aborted fetuses in the present research, well below the 30.8% isolated from Sistan and Baluchestan province, southeastern Iran (Hosein Abadi et al. 2019). A survey was performed in 2017 on 70 sheep flocks that suspected contagious agalactia from 10 provinces of Iran (Shamsaddini Bafti et al. 2017). The results showed that 36% of the animals had *Mycoplasma* infection. However, only 6% of them were related to *M. agalactiae*. Because mycoplasmas other than *M. agalactiae* can also cause abortion, the *M. agalactiae* vaccine may not be totally effective (Hosein Abadi et al. 2019). Differences found among the results of abortion agents in mentioned studies might be due to the diagnostic techniques used, methods and times of sampling, infection rate, different control strategies of each country such as vaccines, use of antibiotics, farmer training, quality of available vaccines and breeding type.

*Toxoplasma* infection was found in aborted fetuses in this survey. The overall seroprevalence rate of this infection among Iranian sheep was estimated to be 31%, and the infection rate in sheep over 1-year-old was 2.4 times more than in younger ones (Sharif et al. 2015). Numerous studies indicate high seroprevalence of *Toxoplasma* infection among sheep in Iran (Hamzavi et al. 2007; Asgari et al. 2013), France (Dumètre et al. 2006), and Turkey (Yildiz et al. 2014). In contrast, other studies that have identified *T. gondii* as an abortion agent have reported low rates (Razmi et al. 2010; Rassouli et al. 2011; Habibi et al. 2012).

Most surveys in the world have not evaluated the viral causes of abortion in small ruminant flocks except for some, which have reported a variety of results (Behzadi Shahrbabak 2019; Alemayehu et al. 2021). Border disease is considered endemic in small ruminants in Iran, and our results showed that 0.5% of fetuses were infected with the BDV. In the research of Kaleibar et al. (2014), carried out in Iran, which was conducted among suspected ewes with a history of reproductive failure, 11.3% were positive for the genome of the Border virus. Likewise, limited virological studies have been performed to determine the sheep abortion due to BDV. According to Mokhtari and Manshoori, 9% of aborted fetuses were positive for BDV using the RT-PCR test in Iran (Mokhtari and Manshoori. 2018). However, Hemmatzadeh et al. did not report any positive cases of BDV in free-ranging wild ruminants of Iran (Hemmatzadeh et al. 2016). According to our findings, 1.9% of aborted fetuses were infected with the Bluetongue virus (BTV). Different serological studies have been performed to detect BTV antibodies among sheep in Iran and other countries, showing different rates of seroprevalence (Lundervold et al. 2003; Ravishankar et al. 2005; Gür. 2008; Khezri and Azimi 2013; Abera et al. 2018; Elmahi et al. 2020). Cattle infected with BVDV and BTV can infect sheep, and in rural areas of Iran and other countries where pregnant sheep and cattle are reared in close contact, this can be an important source of infection transmission (Radostits and Gay 2017).

This survey identified infectious agents such as *E. coli* and other bacteria in low abortion rate flocks. When bacteria not generally related to ovine abortion were isolated in pure culture from fetal tissues, the isolated microbe was considered the abortion agent. These factors mainly cause sporadic abortions (Hassani-Tabatabaei and Firouzi 2005). Previous research indicates that endotoxins released from gram-negative bacteria such as *E.coli* and *Salmonella* can cause abortion due to prostaglandins release (Schlafer et al. 1994; Youngquist and Threlfall 2006). On the other hand, *E.coli* and other bacteria involved in sporadic abortions can likely be considered opportunists (Kirkbride 1993). In a survey performed in the Netherlands, 5% of sheep abortions among 98 ovine fetuses were reported due to *E. coli* (Van Engelen et al. 2014). Hay and silage storage are not well done in traditional rearing systems; consequently, feeding mouldy hay or poor-quality silage to pregnant ewes increases the risk of abortion due to mycotoxins and fungi (Radostits and Gay 2017).

Non-infectious agents rarely cause an abortion rate higher than 2% in the flock, and in abortion outbreaks with higher rates, an infectious agent is likely the cause (Noakes et al. 2018). Our findings revealed that in flocks with less than 2% abortion, only 2.8% of cases were caused by infectious agents. In addition, congenital defects, pregnancy toxaemia, trauma, and Vit E/selenium deficiency were 0.4%, 1.2%, 1.3% and 0.9% of flock abortion causes, respectively. Unlike goats, pregnancy toxaemia is not common among indigenous Iranian ewes for two reasons: First, their litter size is 1 to 1.2, and secondly, they have fat tails, and they can metabolise this fat storage in an energy crisis, reducing the risk of pregnancy toxaemia. Nevertheless, most regions in Iran are generally Se deficient (Mohri et al. 2011), and despite the direct effect of vitamin E as an antioxidant being obvious (McDowell et al. 1996), some reports on the impact of vitamin E on lamb survival are vague. According to the research of Dønnema et al., there was no effect on the stillbirth rate for ewes having ≤ 2 lambs (Dønnem et al. 2015). Other researchers also found no effect in lamb mortality when supplementing vitamin E in the last weeks of pregnancy (Merrell 1998; Daniels et al. 2000). A survey of abortion factors in Switzerland did not report Vitamin E/Selenium deficiency in aborted sheep fetuses, and in their report, only 2% of aborted kids were for this reason (Chanton-Greutmann et al. 2002).

A substantial number of fetuses in flocks with less than a 10% of abortion rate were undiagnosed in our survey. Unbalanced feed, environmental, hormonal and toxic stressors can affect the ability of the pregnant animals to sustain their fetus and frequently results in no identifiable abortion agents (Clothier and Anderson. 2016). Due to a lack of references on fetal concentrations of minerals, confirmation of mineral imbalances is severely limited, whereas toxic agents responsible for abortion may not be present once tissues are examined (Anderson 2007). Enhanced diagnostic methods for non-infectious agents would increase the likelihood of obtaining a definite result in such cases.

According to multivariate binary logistic regression analysis, the gestational age of aborted fetuses was related to abortion. The majority of aborted fetuses were > 3 months when fetal demands on the ewe are most significant. This is due to two main reasons: Firstly, the primary abortion agents cause abortions during late pregnancy. Secondly, early abortions do not attract the attention of the farmers. Then, since ewes are still in the breeding season, they return to estrus and may become pregnant again (Hamedi et al. 2020; Esmaeili et al. 2021). Likewise, this survey revealed a significant correlation between abortion and lack of mineral supplementation in animal feed. Mineral supplementation can enhance the health condition of animals and is necessary to stimulate an immune response (Suttle 2010). Similar research indicated a significant correlation between *C. abortus* infection and mineral supplementation in small ruminant flocks of Iran (Esmaeili et al. 2021). Further, mineral supplementation helps prevent or reduce the incidence of abortion in sheep (Aytekin and Aypak 2011). Contrary to our findings, some other reports mentioned no relationship between mineral/concentrate supplementation and abortion in sheep flocks (Hidiroglou 1979; Pond and Wallace 1986; Naziroğlu et al. 1998).

This survey revealed that farmers who vaccinated their flocks according to the IVO's schedule had fewer abortion rates. It should be noted that this is not only because of vaccines but also because of better rearing management in these flocks. Among the various infectious causes obtained in this survey, only brucellosis and contagious agalactia commercial vaccines are available in Iran.

The history of Rev-1 vaccination against *B. melitensis* did not show a relationship with abortion in multivariate binary logistic regression analysis. Vaccination with Full dose (FD) Rev-1 at 3 to 8 months of age confers an immunity that lasts 2.5 years in sheep (Radostits and Gay 2017). FD Rev-1 vaccine has been used in Iranian flocks since 1963, and from 2003 to 2013, a reduced dose (RD) Rev-1 was included in the national control program for adult sheep (Esmaeili et al. 2012b). Since the RD Rev-1 vaccine was removed from the control program, the immunity duration of FD Rev-1 is shorter than the reproductive life of ewes (Esmaeili et al. 2012a). Therefore, there are still abortions due to brucellosis in sheep flocks of Iran.

Analyzing multivariable logistic regression, the history of stillbirth is a risk factor for abortion in Iranian sheep flocks. Since most infectious causes of abortion can also cause stillbirth (Youngquist and Threlfall 2006), the presence of stillbirth is expected in these flocks. In accordance with our results, the history of stillbirths in the flock was recognised as an abortion risk factor in previous investigations (Dechicha et al. 2020; Esmaeili et al. 2021).

Various studies have evaluated the risk factors for the most relevant abortion agents in sheep flocks and reported a variety of results. In line with our results, they showed no significant association between abortion and flock size, type of farming, grazing system and contact with other flocks (Kardjadj et al. 2016). In contrast to our results, another study suggests a significant association between abortion and flock size, but their flock size differed (< 15 and > 30) (Gebretensay et al. 2019). Our results demonstrate that the presence of cattle and dogs was not identified as a risk factor for abortion, as was reported in other studies in Algeria and Ethiopia (Gebremedhin et al. 2013; Dechicha et al. 2020).

In conclusion, the results obtained in the present survey revealed that multiple agents are involved in abortion among Iranian sheep flocks (Table 2). The use of vaccines in the control of reproductive diseases of sheep is an essential part of any flock health program and one of the main requirements in intensive systems. Given that most sheep farming in Iran is traditional, any abortion control plan is doomed to failure regardless of the farmers' training.

## Data Availability

The data supporting this study's findings are available from the corresponding author upon reasonable request.
